# News and Notes

**Published:** 1912-09

**Authors:** 


### NEWS AND NOTES

### SECOND TRIENNIAL MEETING OF THE NATIONAL ASSOCIATION FOR THE STUDY OF PELLAGRA. COLUMBIA, S. C., OCTOBER 3 and 4, 1912.

PRELIMINARY PROGRAMME.

Assembly Hall,
State Hospital for the Insane,
October 3, 10:30 A. M.
Invocation.
Addresses of Welcome.
Responses.
Address of President.
Appointment of Committees.
Registration of Members.

ASSEMBLY HALL, STATE HOSPITAL, 2:30 P. M.
ETIOLOGY, EPIDEMIOLOGY AND STATISTICS.

1. "A Preliminary Report on the Epidemiology of Pellagra."
   By the Thompson-McFadden Pellagra Commission.
   Dr. J. F. Siler, Captain, Medical Corps, U. S. Army.
   Dr. P. E. Garrison, Assistant Surgeon, U. S. Navy.
   Dr. W. J. MacNeal, Assistant Director of Laboratories.
2. "The Prevalence and Geographic Distribution of Pellagra in the United States."
   Dr. C. H. Lavinder, Passed Assistant Surgeon, U. S. Public Health Service.
3. "Epidemiology of Pellagra in the United States."
   Dr. R. M. Grimm, Assistant Surgeon, U. S. Public Health Service.
4. "The Practical Handling of the Spoiled Corn Problem."
   Hon. E. J. Watson, Commissioner of Agriculture, Columbia.
5. "One of the Possible Factors in the Causation of Pellagra."
   Allan H. Jennings and W. V. King, Bureau of Entomology, Department of Agriculture, Washington

6. "The Sandfly and Pellagra."
Professor S. J. Hunter, State Entomologist, Lawrence, Kansas.

7. "Etiology of Pellagra."
Professor G. Antonini, Director, Insane Asylum of the Province of Milan, Mombello, Italy.

8. "Studies in Pellagra."
Professor Victor Babes, Pathological Institute, Bucharest, Rumania.

LOCAL HISTORY AND DIAGNOSIS OF PELLAGRA.

9. "Pellagra in Rhode Island."
Dr. A. H. Harrington, Superintendent, State Hospital, Howard, R. I.

10. "Pellagra in the District of Columbia."
Dr. H. H. Hazen, Clinical Professor of Dermatology, Howard University, Washington, D. C.

11. "The Differential Diagnosis of the Cutaneous Manifestations of Pellagra with the Report of Fifteen Cases Occurring in Missouri."
Drs. M. F. Engman and W. H. Mook, St. Louis.

12. "The Geographical Distribution of Pellagra in North Carolina and its Significance."
Dr. E. J. Wood, Wilmington.

13. "History of Pellagra in the State of New York."
Dr. F. C. Curtis, Consulting Dermatologist. State Board of Health, Albany.

14. "Some Observations in Two Hundred and Seventy-Six Cases of Pellagra Reported to the Tennessee State Board of Health."
Dr. J. C. Brooks, Chattanooga.

15. "Pellagra in Texas."
Dr. H. K. Beall, Member State Board of Health, Fort Worth.

15a. "Pellagra in the Canal Zone."
Dr. W. E. Deeks, Isthmian Canal Commission, Canal Zone.

OCTOBER 3D, 8:30 P. M., COLUMBIA THEATRE.

16. Address—Surgeon General Rupert Blue, U. S. Public Health Service.

OCTOBER 4TH, 10:30 A. M., ASSEMBLY HALL, STATE HOSPITAL.

LABORATORY INVESTIGATIONS OF PELLAGRA.

17. "Bacteriological Researches in Pellagra."
Dr. Eugenio Bravetta, First Assistant Physician, Insane Asylum of the Province of Milan, Mombello, Italy.

18. "Studies of the Nervous Histo-Pathology of Pellagra."
Drs. H. Douglas Singer and L. J. Pollock, State Psychopathic Institute, Hospital, Ill.

19. "The Experimental Production of Lesions Resembling Pellagra."
Dr. Herman M. Adler, Psychopathic Department, State Hospital, Boston.

20. "Intestinal Parasites of Pellagrins and Non-Pellagrins."
Dr. Ernest Cooper, Pathologist, State Hospital, Columbia.

21. "The Specific Fluorescent Substances Present in the Blood Serum of Pellagrins."
Dr. A. D. Hirschfelder, Baltimore.

21a. "The Chemistry of Spoiled Corn and Its Possible Relation to Pellagra.
Dr. H. S. Reed, Plant Pathologist, Agricultural Experiment Station, Blacksburg, Va.

CLINICAL FEATURES OF PELLAGRA.

22. "Early and Undeveloped Cases of Pellagra."
Dr. Beverly R. Tucker, Richmond.

23. "The Importance of Mild Cases of Pellagra in the General Consideration of the Subject."
Dr. C. C. Bass, Assistant Professor of Tropical Medicine and Clinical Medicine, Tulane University, New Orleans.

24. "The Analogies of Pellagra."
Dr. Stewart R. Roberts, Associate Professor of the Practice of Medicine, College of Physicians and Surgeons, Atlanta.

25. "Etiologic Factors in Recurrent Attacks of Pellagra."
Dr. Geo. C. Mizell, Atlanta.

26. "Clinical and Pathological Notes on Pellagra."
Dr. C. W. G. Rohrer, Department of Heatlh, Baltimore.

27. "A Case of Beriberi With Erythema Resembling Pellagra."
Dr. Robert Wilson, Jr., Chairman, South Carolina State Board of Health, Charleston.

28. "The Co-existence of Pellagra and Beriberi."
Dr. Eleanora B. Saunders, State Hospital, Columbia.

29. Pellagra Among Children in Orphanages."
Dr. H. W. Rice, President Columbia Medical Society and Acting Physician to the Epworth Orphanage, Columbia.

30. "The Eye Symptoms of Pellagra."
A Second Report.
Dr. E. M. Whaley, Oculist, State Hospital, Columbia.

31. "Facies Pellagrosa."
Dr. Bernard Wolff, Clinical Professor of Skin Diseases, College of Physicians and Surgeons, Atlanta.

32. "The Mental Symptoms of Pellagra."
Dr. Roy M. Van Wort, New Orleans.

ASSEMBLY HALL, STATE HOSPITAL, 2:30 P. M.

TREATMENT OF PELLAGRA.

33. "The Treatment of Pellagra: Its Present Status."
Dr. G. M. Niles, Professor of Gastro-Enterology and Therapeutics, Atlanta School of Medicine, Atlanta.

34. "The Treatment of Pellagra."
Dr. J. J. Watson, Columbia.

35. "Pellagra: Its Treatment."
Dr. E. H. Bowling, Durham, N. C.

36. "Results of Transfusion in Thirty-Five Cases of Pellagra."
Dr. H. P. Cole, Mobile.

37. "The Relative Value of Soamin and Salvarsan in the Specific Treatment of Pellagra."
Dr. E. H. Martin, Hot Springs, Ark.

MISCELLANEOUS ASPECTS OF PELLAGRA.

38. "Is Pellagra Communicable?"
Dr. J. A. Hayne, Secretary and Health Officer, State Board of Health, Columbia.

39. "Medico-Legal Aspects of Pellagra."
Dr. J. W. Babcock, State Hospital, Columbia.

40. "Report of Dental Findings in Pellagrins."
Dr. S. F. Killingsworth, Dentist, State Hospital, Columbia.

41. "The Sociological Side of Pellagra."
L. B. Myers, General Secretary, Associated Charities, Charlotte.

ASSEMBLY HALL, STATE HOSPITAL, 8:30 P. M.

42. Lantern Slides of Pellagra.

Papers have also been promised, but the subjects not yet announced, by:

Dr. Carl L. Alsberg, Chemical Biologist, Bureau of Plant Industry, Department of Agriculture, Washington, D. C.

Dr. F. M. Sandwith, London England.

Dr. G. A. Zeller, Superintendent, State Hospital, Peoria, Ill.

Dr. L. W. Sambon, London, England.

Dr. J. N. Thomas, Superintendent, State Hospital, Pineville, La.

Dr. B. L. Eiker, President, State Board of Health, Leon, Iowa.

Dr. Robt. S. Carroll, Asheville, N. C.

Dr. W. D. Partlow, Bryce Hospital, Tuscaloosa, Ala.

Dr. N. M. Moore, Augusta.

Dr. D. J. Williams, The Asylum, Kingston, Jamaica.

Dr. C. J. Manning, Superintendent, Rosemont, Barbados.

Dr. J. W. Mobley, State Sanitarium, Milledgville, Ga.

Dr. G. F. Gaumer, Tacubaya, Yucatan, Mexico.

Dr. C. J. Gaumer, Izamal, Mexico.

Dr J. A. Mackintosh, Wilson City, Abacco, Bahama Islands.

Dr. E. Bates Block, Atlanta.

Dr. John E. S. Davidson, Charlotte.

Dr. W. O. Nisbet, Charlotte.

Dr. Isadore Dyer, New Orleans.
Dr. John McCampbell, Morganton, N. C.
Dr. R. T. Dorsey, Atlanta.
Dr. Jean Nicolaidi, Paris, France.
Dr. J. H. Taylor, Columbia.
Dr. M L. Ravitch, Louisville, Ky.
Dr. H. F. Harris, Secretary, State Board of Health, Atlanta.
Frederick Knab, U. S. National Museum, Washington.
Dr. Minnie C. Dunlap, State Hospital, Lexington, Ky.

Dr. Myers, Professor of Physiological Chemistry, Post-Graduate Medical School, New York City.

Dr. Hillman, New York City.

Dr. I. H. Goss, Athens, Ga.

In addition, papers are expected from authorities in England, Italy, France, Austria, Spain, Rumania, Algiers, Egypt, the Philippine Islands, the Panama Canal Zone, Mexico, Brazil, the Argentine Republic, Cuba, Porto Rico and the Bahama Islands.

As will be seen, every effort is being made by the committees to enlist the interest of pellagra specialists all over the world, so that the meeting will be international in character.

A cordial invitation is extended to all persons interested in pellagra to join the Association, the membership of which is not restricted to physicians and scientists, but extended to laymen as well.

A demonstration of pellagra cases will take place at the close of each day's session. It is especially requested that cases be presented by their family physicians. Opportunity to study the cases in the wards of the State Hospital will also be afforded.

Physicians having photographs of pellagrins, which illustrate different phases of the disease, are requested to forward copies to the committee so that the lantern slide exhibition may be as instructive and complete as possible.

The general committee appointed by the South Carolina Medical Association to represent it in the management and arrangements for the Conference are: Drs. J. A. Hayne, Chairman, Columbia; Robert Wilson, Jr., Charleston; D. B. Frontis, Ridge

Spring; G. A. Neuffer, Abbeville; Theodore Maddox, Union; J. J. Watson, Columbia; H. E. McConnell, Chester, and A. M. Brailsford, Mullins, and Hon. E. J. Watson, Columbia.

The executive committee, consisting of Drs. J. W. Babcock, J. A. Hayne and J. J. Watson, Columbia, S. C., will be pleased to receive further suggestions relative to the final program.

The committee of arrangements, composed of Drs. H. W. Rice, C. F. Williams, and J. LaB. Ward, Columbia, S. C., will give information about hotels, etc., upon application.

The following are hotels in Columbia with their rates per day:

Imperial, 100 rooms, American, $2.50 to $4.00.
Colonia, 100 rooms, American, $3.00 to $5.00.
Jerome, 100 rooms, American, $2.50 to $4.00.
Columbia, 100 rooms, American, $2.50 to $3.50.
Gresham, 100 rooms, European, $1.00 to $2.00.
Caldwell, 30 rooms, European, $1.00.
Albert, 75 rooms, European, $1.00 to $1.50.
Woodward, 20 rooms, European, 75c. to $1.00.
Tavern, 25 rooms, European, $1.00.
Huiet, 20 rooms, American, $1.50.
McCullough, 30 rooms, American, $2.00.

All these hotels are on Main Street except the Colonia, which is located on Hampton Avenue. A Bureau of Information will be provided with offices on Main street, at the State Board of Health and the State Hospital for the Insane.

---

### AMERICAN PROCTOLOGIC SOCIETY.
### FOURTEENTH ANNUAL MEETING, HELD AT ATLANTIC CITY, N. J., JUNE 3 and 4, 1912.

*Post-Operative Care of Rectal Cases.—By Wm. M. Beach, M. D., of Pittsburgh, Pa.*

*(Continued from Last Issue.)*

*Valvotomy.—By George B. Evans, M. D., of Dayton, Ohio.*

Valvotomy as a factor for the relief of proctitis.

Valvotomy as a factor for the relief of obstipation and constipation.

Valvotomy as a factor for the relief of distinct and isolated ulceration of the distal side and adjacent to the valve.

Valvotomy as a factor in the elimination of bladder and prostatic symptoms reflexly.

The location of the valves. Every case of valvular trouble is accompanied by pathological changes in the valves, and if in the valve, then in the adjacent tissues.

Valvular obstruction aer causative factors in the production of obstipation in a large per cent. of our cases.

Valvular obstruction are causative factors in the production obstipation and constipation, but often causes reflex and neurasthenic symptoms to disappear; frequently ameliorates and even cures proctitis, and by virtue of the drainage it secures, lessens the tendency to toxemia from intestinal origin.

---

*Multiple Adenomata of the Rectum—A Report of a Case with Symptomatic Relief by Simple Remedies.—By E. H. Terrell, M. D., of Richmond, Va.*

This article was a report of a case of multiple adenomata of the rectum and sigmoid, in a patient 42 years of age, who had been suffering for the past five years. He had frequent stools with mucus, some blood and a great deal of tenesmus. He was having from eight to ten stools daily. He suffered considerable pain throughout the abdomen. Examination showed numerous small tumors scattered through the rectum and sigmoid. Microscopic examination showed these growths to be adenomatas. The bowel was intensely inflamed and contained many ulcers. Under irrigation of the bowel with boric acid and the administration by mouth of castor oil and aromatic syrup of rhubarb, improvement was almost immediate. In three and a half months the patient had gained seven and a half pounds, and was comparatively comfortable. The tumors were reduced in size and the ulcers gradually disappeared. While the adenomatas are still present, the patient is symptomatically cured.

Dr. Terrell emphasized the value of the administration of equal parts of rhubarb and castor oil, and thinks that in simple ulceration of the rectum, this treatment alone is almost a specific.

He calls attention to many reports of cases in which adenomatas of the rectum are supposed to disappear, and points out that this condition must be merely a hyperplasia with inflammation and not true tumors, for the latter are permanent. As regards the predisposition of adenomata to become cancerous, he called attention to the fact that these tumors are benign and are consequently composed of mature tissue, so they can not themselves become immature tissue,—which is malignancy. Instead of malignant degeneration, it is likely that matrices of immature tissue have also been deposited where so many matrices of mature tissue are found, and the growth of the adenomata, with the accompanying inflammation and ulceration, stimulates these immature matrices to develop into cancer; or, else, immature matrices are formed from the ulcers, just as they develop from ulcers, in cancer of the stomach. The simple treatment which he proposed not only relieves the patient's symptoms, but by lessening the inflammation and curing the ulcers, it, also, decreases the chances for subsequent malignancy.

---

*Pigmentation of the Rectum and Sigmoid.—By Jerome M. Lynch, M. D., of New York City, N. Y.*

The paper was based on six cases which came under the observation of Dr. Tuttle and himself. He divided pigmentation into Exogenous and Endogenous.

Endogenous—(Hemocromatosis; Pseudomelanosis; Melanosis.)

Exogenous—(Pigmentation due to Chemicals; or Metallic Pigmentation.)

He proceedd to discuss the origin of pigment, and considered Pick's theory concerning the origin of melanosis in pigmentation of the large bowel particularly interesting.

It is as follows:

That the connective tissue cells possess an enzyme tyrosinase which converts aromatic bodies into melanin.

After having reviewed the subject of pigmentation, he reaches the following conclusions:

That hemochromatosis is of bacterial origin; that the extent of the disease is dependent upon the severity of the infection; that the probable source of infection is the intestinal tract, possibly starting as an intestinal putrefaction; that this intestinal purefaction lowers the vitality of the tissues, and thereby the cells of the mucous membrane lose their protective properties, consequently bacteria find ready access to the portal circulation. As a result of this the chromogenic function of the liver is interfered with, consequently the liver becomes surfeited with pigment, and is not capable of abstracting the iron from the hemoglobin, with the result that an excessive amount of pigment is circulating in the blood. That the cells of the intestine probably have a selective action for these pigments, and as a consequence they are deposited in the tissue. That local hemochromatosis may be due to repeated local hemorrhages, followed by infection, and that as a result of this infection the bacteria cause a hemolysis of the blood, forming pigment which resembles hemosiderin, hemotoiden and hemofucin. That these pigments may or may not give a reaction for iron.

So little is known about the structural products of melanin or melanoids, that it is difficult to give the origin of these bodies. Undoubtedly there are several distinct melanins, and their origins must also be distinct. The ferruginous melanins should be considered as originating from blood pigment until further research proves the contrary. Most melanins yield endol, scatol and pyrol. It has been proved that the enzyme tyrosinase is present in the tissues and further that this enzyme is capable of converting aromatic bodies into melanin.

That Pick's theory is ingenious and worthy of consideration, we admit; but there are points that are hard to reconcile with our present conception of cellular activity.

It is hard to understand why he should attribute to connective tissues a highly specialized function; that this is directly opposed to all our preconceived notions of this cell, which heretofore has been supposed to have only one function—that of binding other tissues together, with an enzyme of its own nourishment.

It is a well known fact that the cells of the mucous membrane have the power of neutralizing poisons and converting them into insoluble compounds. In the case of mercury and lead they are converted into sulphides, and as a result of this change, blackening of the tissues, somewhat resembling melanin, takes place.

Drs. Tuttle and Lynch believe that the cases reported by the English observers, were as stated, and should not have been included in Pick's series. Further, that as a result of the action of sulphate of hydrogen on the iron pigments, an insoluble sulphide of iron is formed, blackening of the tissues takes place. This is a separate and distinct form of pigmentation, and should not be confounded with melanosis.

---

*Observations upon the Relationship of Tuberculosis to Peri-Rectal Suppurations.—By Collier F. Martin, M. D., of Philadelphia, Pa.*

The author has found pulmonary tuberculosis so frequently associated with his cases of peri-rectal suppuration that he determined to report a consecutive series of cases, with findings.

The report comprises 376 consecutive cases, 75 per cent. being males, and ranging in age from 7 months to 87 years. The majority of these cases (322) occurred in the most active period of life, from 20 to 60 years.

He divided his cases into four major groups; the actively tubercular (144 cases), the chronically tubercular (68 cases), the phthisenoid (20 cases), and those patients in apparently good health (55) cases. This would indicate that at least 212 cases, or 61 per cent., were cases of known tuberculosis.

There were 309 operations performed on 306 patients, under various anaesthetics; spinal anaesthesia 145 times, ether 54 times, and local and other anaesthetics on the remaining. He chose spinal anaesthesia where no other preference was expressed by the patient or the attending physician, on account of the associated tuberculosis.

Following these cases for the past four years he has traced thirty-seven deaths, of which thirty-four died of active tuberculosis or its complications.

The abscesses or fistulae in most of these cases could not be classified, from their appearance, as being locally tuberculous. Where the tubercle bacillus was easily recovered from the tissues or discharges, there was usually a very active pulmonary infection present.

The writer believes that the usual explanation of the association of pulmonary tuberculosis with rectal suppurations, lies in the fact that any pulmonary lesion, however small or inactive, may so alter the patient's vital processes and so lower the opsonic index, as to make him particularly susceptible to pyrogenic invasion. The same may be said of pyogenic infections in general, but the peculiar anatomic conditions existing in the rectum and its very active physiologic function, makes this a fertile region for external and internal trauma with subsequent inflammation and infection.

Thirty-two of these 94 pruritus cases have been examined bacteriologically by him and all of them showed streptococcic skin infection as the predominating condition.

Traumatism is considered to be the chief active factor in impairing the integrity of the tissues.

The writer emphasized the fact that a careful lung examination should be made in all cases of peri-rectal suppuration. He also made a strong plea for a careful and extended supervision of the patient's general health for a long period after all surgical treatment had been discontinued.

The vital consideration in these cases is not the question as to whether or not the local lesion is tuberculous, but has to do with the presence or absence of active or latent tuberculosis in the patient, and his chances of having good general health after surgical intervention.

---

*Ano-Rectal Diseases Due to Veneral Infection.—By James A. McVeigh, M. D., of Detroit, Mich.*

Venereal disease is an important factor in the etiology of disease in all parts of the human system. Regional relationship of genital organs to anus and rectum render the latter especially prone to this kind of infection. Venereal disease of anus and rectum either direct, through practice of vicious habits, or indirect, or accidental, through extension of infection to these parts from other sources. Less direct infection of this nature in this than in foreign countries. Gonorrhoea, Chanchroid and Syphilis, the principal veneral factors in ano-rectal disease. Description of symptoms, diagnosis and treatment of these conditions when appearing in disease of the rectum and anus. Report of a case.

---

*Further Observations on Pruritus Ani: Its Probable Etiologic Factor Based upon Original Research.—By Dwight H. Murray, M. D., of Syracuse, N. Y.*

This paper was a continuation of the work that he has been engaged in for the past two years and which he presented to the American Proctologic Society, at the Los Angeles meeting, in 1911.

From his experiences, since discovering that a skin infection is the most important factor in pruritus ani, he believes that we are now in a position to state that there may be two varieties of pruritus ani; one that may be coincident with some of the diseases of the rectum and in which the skin infection is not present. He designates this form as Pruritus Ani Simplex; the variety which is chronic in its character, and in which the skin infection is present, he designates as Coccigenous Pruritus Ani.

He states that he is continually seeing patients who have all varieties of rectal diseases, including chronic diarrhea and proctitis, in many of which there is a leakage of moisure upon the anal skin; in very few of these cases does he find pruritus ani, and he believes that when it is present, it is coincident rather than having been caused by these discharges occurring in various rectal diseases.

He gives a resume of an examination of 900 consecutive cases, in which he finds 490 cases of constipation, 369 of hemorrhoids, and 94 of pruritus ani. Of the 94 cases which gaves a history of pruritus ani, he finds that 5.5% of the 900 cases examined who had pruritus ani were constipated; 2.3% had hemorrhoids; 1.2% had some form of anal growth; 2.2% had ulceration; 2.5% had diseased crypts; 1.3% had hypertrophied papillae;

.03% had polypi; .03% had fistulae. He believes that the relatively small percentage of each of these conditions that were present in the pruritus ani cases, shows that they were coincidental when present and could not be classed as pruritus ani.

He believes that the excess moisture and the infiltrated condition of the skin in these cases is due to the low grade inflammation caused by skin infection and is not the result of moisture coming from the inside of the anal canal.

He presented photographs of petri-plates, of a typical case, showing the immense numbers of streptococci at the time of the first examination; another photograph of the same case, showing that streptococci were not present in the culture taken from the anal canal, and another photograph of a petri-plate, of the same case, after four months of treatment (one month after itching had ceased), in which last photograph no streptococci were present.

He gives a report of his technic in greater detail than in last year's paper, because he has found that the last year's report was not understood by some physicians who had employed his method.

From some reports received he believes that stock vaccines will not give good results because they are made of a different branch of the streptococcic family than the one causing pruritus ani.

He gives detailed reports of the cases treated, both of the first and second series, showing very marked improvement in all of the cases and cures, so far as present conditions are concerned, of others.

He presented a series of twelve control cases, having a variety of rectal diseases, that are usually given in text-books as causes of pruitus ani, none of which had the disease nor did they show a skin infection.

He said that the conclusions of the first year's work still hold true, and he gave the conclusions of his second year's work as follows:

1st. It is shown by the nine hundred consecutive cases of rectal diseases that constipation and hemorrhoids, or any lesion, are coincidental, or may be predisposing, but not the exciting cause of pruritus ani.

2nd. Even when there is a discharge of pus or other moisture on the skin about the anus it is not the actual cause of pruritus ani, unless there is a streptococcic or other infection of the skin. They may exist together, but are then only a coincidence.

3rd. All investigators, in making cultures, should use in addition to the hard media, the liquid media and Gordon's series of carbohydrates, if they wish to differentiate the streptococci and other bacteria.

4th. Avoid excessive reaction.

5th. Use small initial doses.

6th. Give subsequent injections only after the previous reaction has completely subsided.

7th. He suggests the following change in the nomenclature of pruritus ani, by recognizing two varieties: Pruritus Ani Simplex and Pruritus Ani Coccigenous.

---

*Colonic Dilatation (Congenital and Acquired) As a Factor in Chronic Intestinal Obstruction (Obstipation).—By Samuel G. Gant, M. D., of New York City, N. Y.*

The author stated that his experience warrants the belief that both acquired and congenital (Hirschprung's) dilatation of the colon is fairly common, and that they respond satisfactorily to treatment (usually surgical). He said that non-congenital dilatation of the bowel might result from paresis, gormandizing, digestive disturbances or chronic intestinal obstruction, however caused, and when present, leads to constipation, fecal impaction, distension of the bowel, angulation, twisting and ptosis of the colon. He called attention to the fact that this class of patients suffered much less from intestinal auto-intoxication than persons afflicted with acute constipation. In his cases, the colon completely filled the abdomen, measured from three to many times its normal size, was considerably thickened, characterized by dilatated blood-vessels and closely resembled an enormously hypertrophied stomach—for which is was mistaken in two instances. He mentioned having personally observed seven cases of Hirschsprung's disease and a still greater number of acquired dilatation, wherein the patients had an evacuation every two or three weeks, following purgation and frequent enemata; except in two cases, that of a young boy, who moved his bowels only once in two months, and of a young woman, who succeeded in accomplishing this but four times yearly. He said the chief manifestations of the condition were those of chronic constipation and fecal impaction, plus mal-nutrition, abdominal distension, pot-belly, extraordinary length of time between the movements and very large amount of feces discharged when an evacuation occurred, and that the diagnosis is fairly easy in the presence of the above symptom complex, because, with the aid of inflation and palpation or the assistance of the X-Ray, the size and position of the colon can be defined.

The writer maintained that temporary improvement occasionally follows medication and physical measures, which strengthen the bowel or minimize the effects of autointoxication consequent upon fecal retention, and that the patient may for weeks or years be kept fairly comfortable when given close attention and the bowel is kept open with lubricating oils, laxatives and frequent high enemata, but that a cure is not possible except through one of the following surgical measures, viz:

1. Coloplication.

2. Colopexy.

3. Resection.

4. Intestinal exclusion.

5. Colostomy.

6. Tapping.

He found coloplication effective in both congenital and acquired dilatation, without bowel displacement. Coloplexy proved satisfactory where there was ptosis with moderate dilatation, but in aggravated cases where the bowel was both enormously dilated and markedly ptotic, he advised coloplication and coloplexy, using the infolding sutures for suspensory purposes.

He advised resection of all or part of the colon where it was irretrievably large, displaced or bound down by adhesions, and reported a case where the sigmoid flexure, descending colon and left half of the transverse colon were excised.

Exclusion had proven satisfactory, and he reported five cases treated by dividing the ileum near the cecum and completing the exclusion by ileo-sigmoidostomy.

Colostomy was looked upon with ill-favor, because patients strenuously object to an artificial anus, and, a secondary and dangerous operation is required to re-establish continuity of the intestine.

Tapping, he said, deserved no consideration, because it is unscientific, dangerous and ineffective.

In closing, Dr. Gant said that he frequently combined the above operations with appendicostomy or cecostomy, so that through and through irrigation could be immediately established and the period of convalescence shortened. He also stated that colonic exclusion and colostomy were considerably less dangerous than resection, and were usually effective, since the bowel rapidly contracts after their establishment.

(*To Be Continued.*)

---

### PRINCIPLES OF MEDICAL ETHICS.

### *Chapter I.—The Duties of Physicians to Their Patients.*

THE PHYSICIAN'S RESPONSIBILITY.

SECTION 1.—A profession has for its prime object the service it can render to humanity; reward or financial gain should be a subordinate consideration. The practice of medicine is a profession. In choosing this profession an individual assumes an obligation to conduct himself in accord with its ideals.

SEC. 2.—Patience and delicacy should characterize all the acts of a physician. The confidences concerning individual or domestic life entrusted by a patient to a physician, and the defects of disposition or flaws of character observed in patients during medical attendance should be held as a trust and should never be revealed except when imperatively required by the laws of the state. There are occasions, however, when a physician must determine whether or not his duty to society requires him to take definite action to protect a healthy individual from becoming infected, because the physician has knowledge, obtained through the physician, of a communicable disease to which the healthy individual is about to be exposed. In such a case, the physician should act as he would desire another to act toward one of his own family under like circumstances. Before he determines his course, the physician should know the civil law of his commonwealth concerning privileged communications.

SEC. 3.—A physician should give timely notice of dangerous manifestations of the disease to the friends of the patient. He should neither exaggerate nor minimize the gravity of the patient's condition. He should assure himself that the patient or his friends have such knowledge of the patient's condition as will serve best interests of the patient and the family.

SEC. 4.—A physician is free to choose whom he will serve. He should, however, always respond to any request for his assistance in an emergency or whenever temperate public opinion expects the service. Once having undertaken a case, a physician should not abandon or neglect the patient because the disease is deemed incurable; nor should he withdraw from a case for any reason until a sufficient notice of a desire to be released has been given the patient or his friends to make it possible for them to secure another medical attendant.

### *Chapter II.—The Duties of Physicians to Each Other and to the Profession at Large.*

### ARTICLE I.—DUTIES TO THE PROFESSION.

SECTION 1.—The obligation assumed on entering the profession requires the physician to comport himself as a gentleman and demands that he use every honorable means to uphold the dignity and honor of his vocation, to exalt its standards and to extend its sphere of usefulness. A physician should not base his practice on an exclusive dogma of sectarian system, for "sects are implacable despots; to accept their thralldom is to take away all liberty from one's actions and thought." (Nicon, father of Galen.)

SEC. 2.—In order that the dignity and honor of the medical profession may be upheld, its standards exalted, its sphere of usefulness extended, and the advancement of medical science promoted, a physician should associate himself with medical societies and contribute his time, energy and means in order that these societies may represent the ideals of the profession.

SEC. 3.—A physician should be "an upright man, instructed in the art of healing." Consequently, he must keep himself pure in character and conform to a high standard of morals and must be diligent and conscientious in his studies. "He should also be modest, sober, patient, prompt to do his whole duty without anxiety; pious without going so far as superstition, conducting himself with propriety in his profession and in all the actions of his life." (Hippocrates.)

SEC. 4.—Solicitation of patients by circulars or advertisements, or by personal communications or interviews, not warranted by personal relations, is unprofessional. It is equally unprofessional to procure patients by indirection through solicitors or agents of any kind, or by indirect advertisement, as by furnshing or inspiring newspaper comments concerning cases in which the physician has been or is concerned. All other like self-laudations defy the traditions and lower the tone of any profession and so are intolerable. The most worthy and effective advertisement possible, even for a young physician, and especially with his brother physicians, is the establishment of a well-merited reputation for professional ability and fidelity. This cannot be forced, but must be the outcome of character and conduct. The publication or circulation of simple business cards, being a matter of personal taste or local custom, and sometimes of convenience, is not *per se* improper. As implied, it is unprofessional to disregard local customs or offend recognized ideals in publishing or circulating such cards.

It is unprofessional to promise radical cures; to boast of cures and secret methods of treatment or remedies; to exhibit certificates of skill or of success in the treatment of disease; or to employ any methods to gain the attention of the public for the purpose of obtaining patients.

SEC. 5.—It is unprofessional, for personal profit, to hold patents for surgical instruments or medicines; to accept rebates on prescriptions or surgical appliances, or prerequisites from attendants who aid in the care of patients.

SEC. 6.—It is unprofessional for a physician to assist unqualified persons to evade legal restrictions governing the practice of medicine; it is equally unethical to prescribe or dispense secret medicines or other secret remedial agents, or manufacture or promote their use in any way.

SEC. 7.—Physicians should expose without fear or favor, before the proper medical or legal tribunals, corrupt or dishonest conduct of members of the profession. Every physician should aid in safe-guarding the profession against the admission to its ranks of those who are unfit or unqualified because deficient either in moral character or education.

ARTICLE II.—PROFESSIONAL SERVICES OF PHYSICIANS TO EACH OTHER.

SECTION I.—Experience teaches that it is unwise for a physician to treat members of his own family or himself. Consequently, a physician should always cheerfully and gratuitously respond with his professional services to the call of any physician practicing in his vicinity, or of the immediate family dependents of physicians.

SEC. 3.—When a physician from a distance is called upon to advise another physician or one of his family dependents, and the physician to whom the service is rendered is in easy financial circumstances, a compensation that will at least meet the traveling expenses of the visiting physician should be proffered. When such a service requires an absence from the accustomed field of professional work of the visitor that might reasonably be expected to entail a pecuniary loss, such loss should, in part at least, be provided for in the compensation offered.

SEC. 3.—When a physician or a member of his dependent family is seriously ill, he or his family should select a physician from among his neighboring colleagues to take charge of the case. Other physicians may be associated in the care of the patient as consultants.

ARTICLE III.—DUTIES OF PHYSICIANS IN CONSULTATIONS.

SECTION 1.—In serious illness, especially in doubtful or difficult conditions, the physician should request consultation.

Sec. 2.—In every consultation, the benefit to be derived by the patient is of first importance. All the physicians interested in the case should be frank and candid with the patient and his family. There never is occasion for insincerity, rivalry or envy and these should never be permitted between consultants.

Sec. 3.—It is the duty of a physician, particularly in the instance of a consultation, to be punctual in attendance. When, however, the consultant or the physician in charge is unavoidably delayed, the one who first arrives should wait for the other for a reasonable time, after which the consultation should be considered postponed. When the consultant has come from a distance or when for any reason it will be difficult to meet the physician in charge at another time or if the case is urgent, or if it be the desire of the patient, he may examine the patient, and mail his written opinion, or see that it is delivered under seal, to the physician in charge. Under these conditions, the consultant's conduct must be especially tactful; he must remember that he is framing an opinion without the aid of the physician who has observed the course of the disease.

Sec. 4.—When a patient is sent to one specially skilled in the care of the condition from which he is thought to be suffering, and for any reason it is thought impracticable for the physician in charge of the case to accompany the patient, the physician in charge should send to the consultant by mail, or in the care of the patient under seal, a history of the case, together with the physician's opinion and an outline of the treatment, or so much of this as may be of service to the consultant; and as soon as possible after the case has been seen and studied, the consultant should address the physician in charge and advise him of the results of the consultant's investigation of the case. Both these opinions are confidential and must be so regarded by the consultant and by the physician in charge.

Sec. 5.—After the physicians called in consultation have completed their investigations of the case, they may meet by themselves to discuss conditions and determine the course to be followed in the treatment of the patient. No statement or discussion of the case should take place before the patient or friends, except in the presence of all the physicians attending, or by their common consent; and no opinions or prognostications should be delivered as a result of the deliberations of the consultants, which have not been concurred in by the consultants at their conference.

SEC. 6.—The physician in attendance is in charge of the case and is responsible for the treatment of the patient. Consequently, he may prescribe for the patient at any time and is privileged to vary the mode of treatment outlined and agreed on at a consultation whenever, in his opinion, such a change is warranted. However, at the next consultation, he should state his reasons for departing from the course decided on at the previous conference. When an emergency occurs during the absence of the attending physician, a consultant may provide for the emergency and the subsequent care of the patient until the arrival of the physician in charge, but should do no more than this without the consent of the physician in charge.

SEC. 7.—Should the attending physician and the consultant find it impossible to agree in their views of a case, another consultant should be called to the conference or the first consultant should withdraw. However, since the consultant was employed by the patient in order that his opinion might be obtained, he should be permitted to state the result of his study of the case to the patient, or his next friend in the presence of the physician in charge.

SEC. 8.—When a physician has attended a case as a consultant, he should not become the attendant of the patient during that illness except with the consent of the physician who was in charge at the time of the consultation.

ARTICLE IV.—DUTIES OF PHYSICIANS IN CASES OF INTERFERENCE.

SECTION 1.—The physician, in his intercourse with a patient under the care of another physician should observe the strictest caution and reserve; should give no disingenuous hints relative to the nature and treatment of the patient's disorder; nor should the course of conduct of the physician, directly or indirectly, tend to diminish the trust reposed in the attending physician.

SEC. 2.—A physician should avoid making social calls on those who are under the professional care of other physicians without the knowledge and consent of the attendant. Should such a friendly visit be made, there should be no inquiry relative to the nature of the disease or comment upon the treatment of the case, but the conversation should be on subjects other than the physical condition of the patient.

SEC. 3.—A physician should never take charge of or prescribe for a patient who is under the care of another physician, except in an emergency, until after the other physician has relinquished the case or has been properly dismissed.

SEC. 4.—When a physician does succeed another physician in the charge of a case, he should not make comments on or insinuations regarding the practice of the one who preceded him. Such comments or insinuations tend to lower the esteem of the patient for the medical profession and so react against the critic.

SEC. 5.—When a physician is called in an emergency and finds that he has been sent for because the family attendant is not at hand, or when a physician is asked to see another physician's patient because of an aggravation of the disease, he should provide only for the patient's immediate need and should withdraw from the case on the arrival of the family physician after he has reported the condition found and the treatment administered.

SEC. 6.—When several physicians have been summoned in a case of sudden illness or of accident, the first to arrive should be considered the physician in charge. However, as soon as the exigencies of the case permit, or on the arrival of the acknowledged family attendant or the physician the patient desires to serve him, the first physician should withdraw in favor of the chosen attendant; should the patient or his family wish someone other than the physician known to be the family physician to take charge of the case, the patient should advise the family physician of his desire. When, because of sudden illness or accident, a patient is taken to a hospital, the patient should be returned to the care of his known family physician as soon as the condition of the patient and the circumstances of the case warrant this transfer.

SEC. 7.—When a physician is requested by a colleague to care for a patient during his temporary absence, or when, because of an emergency, he is asked to see a patient of a colleague, the physician should treat the patient in the same manner and with the same delicacy as he would have one of his own patients cared for under similar circumstances. The patient should be returend to the care of the attending physician as soon as possible.

SEC. 8.—When a physician is called to the patient of another physician during the enforced absence of that physician, the patient should be relinquished on the return of the latter.

SEC. 9.—When a physician attends a woman in labor in the absence of another who has been engaged to attend, such physician should resign the patient to the one first engaged, upon his arrival; the physician is entitled to compensation for the professional services he may have rendered.

ARTICLE V.—DIFFERENCES BETWEEN PHYSICIANS.

SECTION 1.—Whenever there arises between physicians a grave difference of opinion which cannot be promptly adjusted, the dispute should be referred for arbitration to a committee of impartial physicians, preferably the Board of Censors of a component county society of the American Medical Association.

ARTICLE VI.—COMPENSATION.

SECTION 1.—The poverty of a patient, the mutual professional obligation of physician and certain public duties should command the gratuitous services of a physician. A physician should also give his services to a selected group of eleemosynary institutions, but institutions endowed by the public, or by the rich, or by societies, and organizations for mutual benefit, or for accident, sickness and life insurance, or for analogous purposes should be accorded no such privileges.

SEC. 2.—It is unprofessional for a physician to dispose of his services under conditions that make it impossible to render adequate service to his patient or which interfere with reasonable competition among the physicians of a community. To do this is detrimental to the public and to the individual physician, and lowers the dignity of the profession.

SEC. 3.—It is detrimental to the public good and degrading to the profession, and therefore unprofessional, to give or receive a commission or to divide a fee for medical advice or surgical treatment, unless the patient or his next friend is fully informed as to the terms of the transaction. The patient should be made to realize that a proper fee should be paid the family physician for the service he renders in determining the surgical or medical treatment suited to the condition, and in advising concerning those best qualified to render any special service that may be required by the patient.

*Chapter III.—The Duties of the Profession to the Public.*

SECTION 1.—Physicians, as good citizens and because their professional training specially qualifies them to render this service, should give advice concerning the public health of the community. They should bear their full part in enforcing its laws and sustaining the institutions that advance the interests of humanity. They should co-operate especially with the proper authorities in the administration of sanitary laws and regulations. They should be ready to counsel the public on subjects relating to sanitary police, public hygiene and legal medicine.

SEC. 2.—Physicians, especially those engaged in public health work, should enlighten the public regarding quarantine regulations; on the location, arrangement and dietaries of hospitals, asylums, schools, prisons and similar institutions; and concerning measures for the prevention of epidemic and contagious diseases. When an epidemic prevails, a physician must continue his labors for the alleviation of the suffering people, without regard to the risk of his own health or life or to financial return. At all times, it is the duty of the physician to notify the properly constituted public health authorities of every case of communicable disease under his care, in accordance wit hthe laws, rules and regulations of the health authorities of the locality in which the patient is.

SEC. 3.—Physicians should warn the public against the devices practiced and the false pretentions made by charlatans which may cause injury to health and loss of life.

Sec. 4.—By legitimate patronage, physicians should recognize and promote the profession of pharmacy; but any pharmacist, unless he assumes to prescribe for the sick, should be denied unless he be qualified as a physician, who assumes to prescribe for the sick, should be denied such countenance and support. Moreover, whenever a druggist or pharmacist dispenses deteriorated or adulterated drugs, or substitutes one remedy for another designated in a prescription, he thereby forfeits all claims to the favorable consideration of the public and physicians.

CONCLUSION.

While the foregoing statements express in a general way the duty of the physician to his patients, to other members of the profession and to the profession at large, as well as the profession to the public, it is not to be supposed that they cover the whole field of medical ethics, or that the physician is not under many duties and obligations besides these herein set forth. In a word, it is incumbent on the physician that under all conditions, his bearing toward patient, the public, and fellow practitioner should be characterized by a gentlemanly deportment and that he constantly should behave toward others as he desires them to deal with him. Finally, thes principles are primarily for the good of the public, and their enforcement should be conducted in such a manner as shall deserve and receive the endorsement of the community.—(Adopted at the 1912 meeting.)

